# The restorative role of annexin A1 at the blood–brain barrier

**DOI:** 10.1186/s12987-016-0043-0

**Published:** 2016-09-21

**Authors:** Simon McArthur, Rodrigo Azevedo Loiola, Elisa Maggioli, Mariella Errede, Daniela Virgintino, Egle Solito

**Affiliations:** 1 Department of Biomedical Sciences, Faculty of Science and Technology, University of Westminster, London, UK; 2 Department of Basic Medical Sciences, Neuroscience and Sensory Organs, Bari University School of Medicine, Bari, Italy; 3 William Harvey Research Institute, School of Medicine and Dentistry, Queen Mary University, London, UK

**Keywords:** Blood–brain barrier, Annexin A1, Inflammation, Metabolism, Multiple sclerosis, Stroke

## Abstract

Annexin A1 is a potent anti-inflammatory molecule that has been extensively studied in the peripheral immune system, but has not as yet been exploited as a therapeutic target/agent. In the last decade, we have undertaken the study of this molecule in the central nervous system (CNS), focusing particularly on the primary interface between the peripheral body and CNS: the blood–brain barrier. In this review, we provide an overview of the role of this molecule in the brain, with a particular emphasis on its functions in the endothelium of the blood–brain barrier, and the protective actions the molecule may exert in neuroinflammatory, neurovascular and metabolic disease. We focus on the possible new therapeutic avenues opened up by an increased understanding of the role of annexin A1 in the CNS vasculature, and its potential for repairing blood–brain barrier damage in disease and aging.

## Blood–brain barrier structure

The blood–brain barrier (BBB) is the major regulator of communication between the peripheral circulation and the brain, acting to protect the central nervous system (CNS) from the damaging consequences of peripheral challenges to homeostasis, such as occur during inflammation and metabolic disease. Given the tight confines of the skull, oedema-induced elevated tissue pressure is highly damaging to neuronal function [1], hence one of the most important functions of the BBB is to limit immune cell extravasation, and to protect brain tissue from the development of localised inflammation. Similarly, the neural environment is highly metabolically active, needing a significant proportion of the body’s energy supply [2], and is thus highly vulnerable to metabolic toxins. As a defence against these, another critical feature of the BBB is the presence of a network of highly efficient efflux transporters in cerebral endothelial cells, acting to extrude metabolic waste products and to prevent potentially toxic molecules from entering the brain [3].

**Fig. 1:**
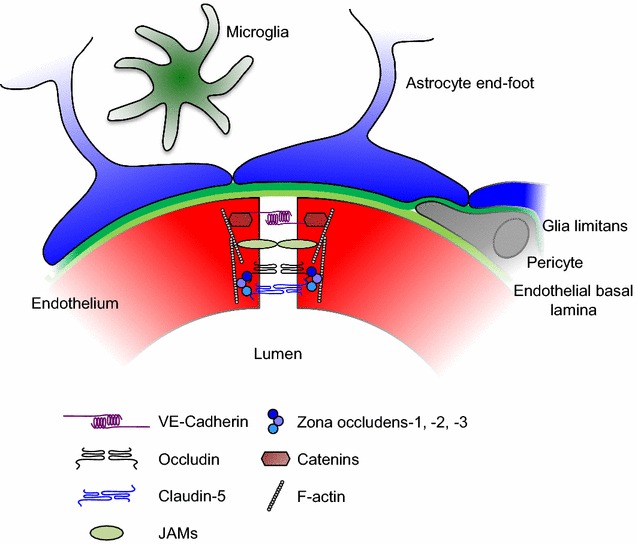
Schematic depiction of the principal molecular and cellular components of the neurovascular unit that regulate inter-endothelial permeability, and thereby provide the foundation of the blood–brain barrier. *JAM* junctional adhesion molecule

The BBB is not an isolated single anatomical structure, but is part of the so called neurovascular unit (NVU), a morpho-functional unit formed by multiple integrated elements of the vessel wall (endothelial cells and pericytes), encircling perivascular astroglia, microglia cells and intervening neuronal terminals [4] (Fig. 1). Central to the barrier function of the NVU are the endothelial cells. These cells are markedly different to other endothelia within the body in that they display interendothelial tight junctions (TJs) organised in an extensive array of junctional strands; a network of close transmembrane protein–protein links that, together with adherens junctions and junctional adhesion molecules, essentially prevent small molecules and invading cells from passing across the vessel wall via a paracellular route [5–7]. A number of different proteins are involved in forming TJs in the brain vasculature, but two of the most important are the molecules occludin and claudin-5 which form homodimeric bridges linking neighbouring cells [8]. These molecules in turn complex with a series of intracellular elements, including the proteins zona occludens-1, -2 and -3 (ZO-1, -2 and -3), which couple to the actin cytoskeleton and help give junctions rigidity [9].

Alongside the paracellular pathway leukocytes migration occurs also by transcellular pathways which occurs in a dynamic interaction between leukocytes protrusions at specific site of the endothelium of the BBB [10].

The critical function of the endothelium is the selective regulation of molecular uptake into the brain parenchyma. Given the strength of inter-endothelial TJs, small molecule entry into the brain is essentially negligible under normal conditions. The brain does, however, require both a significant energy input [2], and the removal of neuronal metabolic waste products to the circulation for elimination through the kidneys. As such, an array of small molecule transport proteins are expressed on the surface of endothelial cells, including transporters for glucose, amino acids, nucleosides and many other cations and anions [7, 11]. In addition to these transporters, receptor-mediated uptake systems exist for the larger biomolecules such as lipoproteins, peptides and protein hormones, permitting the selective entry of molecules like insulin and transferrin into the brain. Complementing these uptake systems, the BBB expresses a range of highly effective ATP-binding cassette (ABC) family efflux transporter systems, most notably P-glycoprotein, breast cancer resistance protein (BCRP), multidrug resistance-associated protein (MRP-1 and MRP-2), which together serve to limit exposure of the CNS to potentially neurotoxic molecules [12], and which unfortunately are also a major barrier to the therapeutic treatment of brain diseases with pharmacological agents [13].

The endothelial cells lie on a complex basal lamina (the equivalent of the basement membrane in peripheral vessels without the *lamina reticularis*) [14], which serves not only to provide physical support to the endothelia, but also includes pericytes and is a further barrier between the circulation and the brain parenchyma [4]. The basal lamina is actually a juxtaposed pair of molecular layers indistinguishable anatomically at the level of microvessels, but originating from endothelial cells and from perivascular astrocytes (parenchymal layer). The two layers are similarly composed of members of four major glycoprotein families: laminins, collagen IV isoforms, nidogens and heparin sulphate proteoglycans, including perlecan and agrin [15]. They can be distinguished however, by their complement of laminin subtypes [16], with the endothelial basement layer containing laminin-411 and -511, whilst the parenchymal one contains laminin-111 and -211 [17].

The basal lamina is not simply a passive substrate but is actively involved in communication across the BBB and in the transfer of nutritional support into the brain parenchyma [18, 19]. The contribution this molecular component of the NVU plays in maintaining BBB integrity has not been fully clarified, but enzymes such as the matrix metalloproteinases that disrupt its structure have been implicated in inappropriate immune cell or parasite entry into the brain [20, 21] and in oedema and haemorrhage during cerebrovascular incidents [22–24].

Pericytes embedded in the basal lamina communicate with microvessel endothelial cells performing important regulatory functions controlling vessel diameter and cerebral blood flow [25, 26], and contributing to BBB integrity. Mice lacking brain pericytes are embryonically lethal, but notably have developing BBBs characterised by abnormal distribution of TJ molecules and enhanced vascular permeability [27], indicating an important role for these cells in either the development, differentiation or maintenance of BBB function [28, 29]. This activity is confirmed by studies of murine models of reduced cerebral pericyte number, with a strong negative correlation existing between brain vessel pericyte coverage and vascular permeability [30], emphasising the importance of these cells, even if the fine details of how they contribute to BBB integrity remain unclear.

Astrocytes, present on the parenchymal side of the vascular basal lamina, are major components of the NVU, with individual astrocytes sending out numerous processes and endfeet that under pericyte-derived guidance cues, surround and ensheath the vessel wall [30]. The astrocyte processes provide considerably more than structural support however, as they not only produce the molecular components of the parenchymal basal lamina limitans [31], but also actively promote TJ formation between endothelial cells [32, 33]. Additionally, astrocytes of the BBB have critical roles in brain–blood transport; they actively regulate water uptake through the major cerebral water channel, aquaporin-4, localized on the plasma membrane of endfeet in contact with the vessel wall [34]. They are also intimately involved in nutrient uptake [35] and play an important role in the removal of neuronal metabolic waste products [36, 37]. Whilst less studied than other components of the neurovascular unit, there is some evidence of a role for microglia in the regulation of BBB integrity, particularly under inflammatory conditions [38]. Evidence in vitro indicates that inflammatory activation of microglia can disrupt endothelial TJs through release of reactive oxygen species and cytokines [39, 40], but whether this occurs in vivo, and the extent to which microglia influence BBB function under normal conditions remains to be investigated.

Together with signalling input from parenchymal neurons [41], the neurovascular unit generates a highly efficient barrier to free communication between the circulation and the brain, whilst permitting the selective uptake of requisite nutrients and water, and enabling the removal of waste products of neuronal metabolism. Whilst this system is indeed highly effective under normal homeostatic challenges, it can be significantly perturbed following the onset of disease and inflammation.

### The blood–brain barrier in inflammation

The BBB is not static, but actively changes and responds to inflammatory challenge, whether originating in the periphery or the brain parenchyma. Numerous inflammatory factors have been shown to enhance BBB permeability, as have recently been reviewed in detail [42–44]. Changes to the BBB reflect both alterations in the permeability barrier to small molecules and, with particular relevance to neurodegenerative conditions such as multiple sclerosis (MS) and Alzheimer’s disease (AD), a loss of the normal restrictions on entry of immune cells into the brain parenchyma through changes in the expression of leukocyte adhesion molecules [45, 46]. The mechanisms underlying these changes are complex, and commonly involve interacting circuits and feedback loops centred on the actions of vasoactive mediators and pro-inflammatory cytokines upon endothelial cells and perivascular astrocytes [33]. For example, bradykinin not only acts via B_2_ receptors on endothelial cells to open TJs [47], but also to induce astrocytic release of interleukin-6, which itself can further enhance endothelial cell permeability [48].

On first examination, enhanced BBB permeability upon exposure to inflammatory stimulation would appear to be maladaptive, but an explanation may lie in consideration of the role the BBB plays in the induction of sickness behaviours. These behaviours, commonly associated with inflammatory disease, include deficits in memory and attention, lethargy and anhedonia, and are thought to provide an adaptive advantage, conserving metabolic energy for the fight against infection/damage [49]. Changes in BBB integrity and consequent access of circulating mediators to the CNS parenchyma are thought to be one of the major communication pathways underlying the induction of these behaviours [50], acting in concert with direct vagal information. Although these behaviours are advantageous in the short term, helping to promote recovery, extended and/or inappropriately severe sickness behaviour can be a major source of morbidity in chronic inflammatory conditions [51, 52].

Evidence for a link between disease-associated enhanced BBB permeability and cognitive impairment has been steadily accruing, both in age-related cognitive decline [51, 53], and in pathologies as diverse as stroke [52, 54], vascular dementia [55, 56], AD [57–59], diabetes mellitus [60] and obesity [61]. Significantly, many of these conditions are associated with peripheral inflammatory activity to a greater or lesser extent; hence developing an understanding of the factors regulating BBB permeability may offer the opportunity to modify the negative cognitive aspects of many inflammatory and neurological conditions.

## Annexin A1, peripheral inflammation and the BBB

**Fig. 2:**
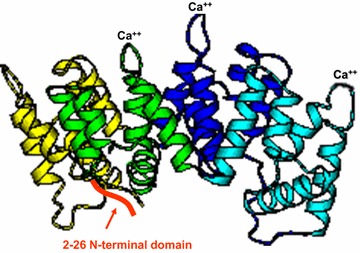
Crystal structure of ANXA1, showing four core Ca^2+^-binding domains, and the N-terminal sequence conferring specificity (2–26 N-terminal domain). Adapted from [65]

For discussion of the general response of the BBB to peripheral and neuroinflammatory challenge we defer to recent comprehensive reports [1, 42, 62–64]; in this paper we will discuss the specific role of one particular mediator known for its peripheral anti-inflammatory/pro-resolution actions, annexin A1 (ANXA1-Fig. 2), with a particular focus on opportunities where it may be used therapeutically to restore damaged BBB function.

The role of ANXA1 as a resolving/protecting molecule in the periphery is well known, as this molecule is a secondary mediator of the anti-inflammatory effects of glucocorticoid hormones [66], preventing leukocyte migration into inflamed tissue [67]. Its role in the CNS has been much less comprehensively studied, however. ANXA1 belongs to the annexin superfamily of proteins (ANXA1–A12) that are near-ubiquitously expressed in eukaryotes from mould to mammals (yeasts being the only major exception) [68]. ANXA1 is a calcium-dependent phospholipid binding protein with a phospholipase A_2_ inhibitory activity. Structurally ANXA1 presents four repeats, three of which contain a Ca^2+^-binding domain (highly conserved among the 13 mammalian annexins) and an N-terminal domain with multiple different phosphorylation sites that regulate its function [69, 70], and which confer its specific anti-inflammatory activity [71]. From an evolutionary point of view the structure of the ANXA1 gene supports the hypothesis that it arose by double duplication of an ancestral single domain gene [72]. Interestingly another member of the family ANXA2 has been recently showed to be involved in miR155 regulation of BBB function [73].

Mechanistically, the auto/paracrine actions of ANXA1 are transduced by its binding to the G protein-coupled receptor formyl peptide receptor 2 (FPR2, also known as the lipoxin A_4_ receptor) [74], which we have shown to be expressed on brain microvascular endothelial cells [75], and by interaction with membrane phospholipids [76]. Numerous intracellular signalling pathways can be activated downstream of ANXA1 binding to FPR2; activation of p38 mitogen-activated protein (MAP) kinase [77], activation of extracellular signal-regulated protein kinase (ERK1/2) and mobilisation of intracellular Ca^2+^ [78], and modification of the actin cytoskeleton through activation of small guanosine triphosphate hydrolase (GTPases) [79, 80] have all been reported.

The role of ANXA1 in the CNS has been debated for several years [81], with its central functions only having been clarified in the last decade. We and others have shown ANXA1 to regulate microglial efferocytosis (non-inflammatory removal of dead cells), and phagocytosis [82–84], supporting a development and anti-inflammatory role, respectively, in the brain. More recently however, we demonstrated an essential homeostatic function of ANXA1, maintaining endothelial TJs in the BBB [80], and repairing the permeabilising effect of systemic lipopolysaccharides (LPS) on the BBB [64]. We propose a dual role for ANXA1 in the CNS vasculature, serving as a homeostatic regulator in normal conditions by promoting BBB integrity, and importantly, acting to prevent and limit the impact of pathological peripheral challenge upon the brain.

## Annexin A1 and the regulation of BBB integrity across the lifespan

ANXA1 is a critical component of the normal BBB. It is expressed by the brain microvascular endothelial cells in close proximity to the plasma membrane and at points of cell–cell contact where it co-localizes with cortex actin microfilaments [75]. Deletion of the ANXA1 gene in null mice is associated with disorganization of the actin cytoskeleton, reduction of stress fibre formation, cell shape changes and a loss of polarity that concludes in the disruption of occludin and VE-Cadherin, findings which indicate that ANXA1 participates in the regulation of BBB permeability through its association with the actin cytoskeleton [75]. In normal health conditions, ANXA1 thus plays a major protective role in the brain through the promotion of BBB integrity. There are two stages during life, however, when BBB function is less than optimal, prenatal development and old age, and there is evidence that changes to ANXA1 expression play a role in both.

**Fig. 3:**
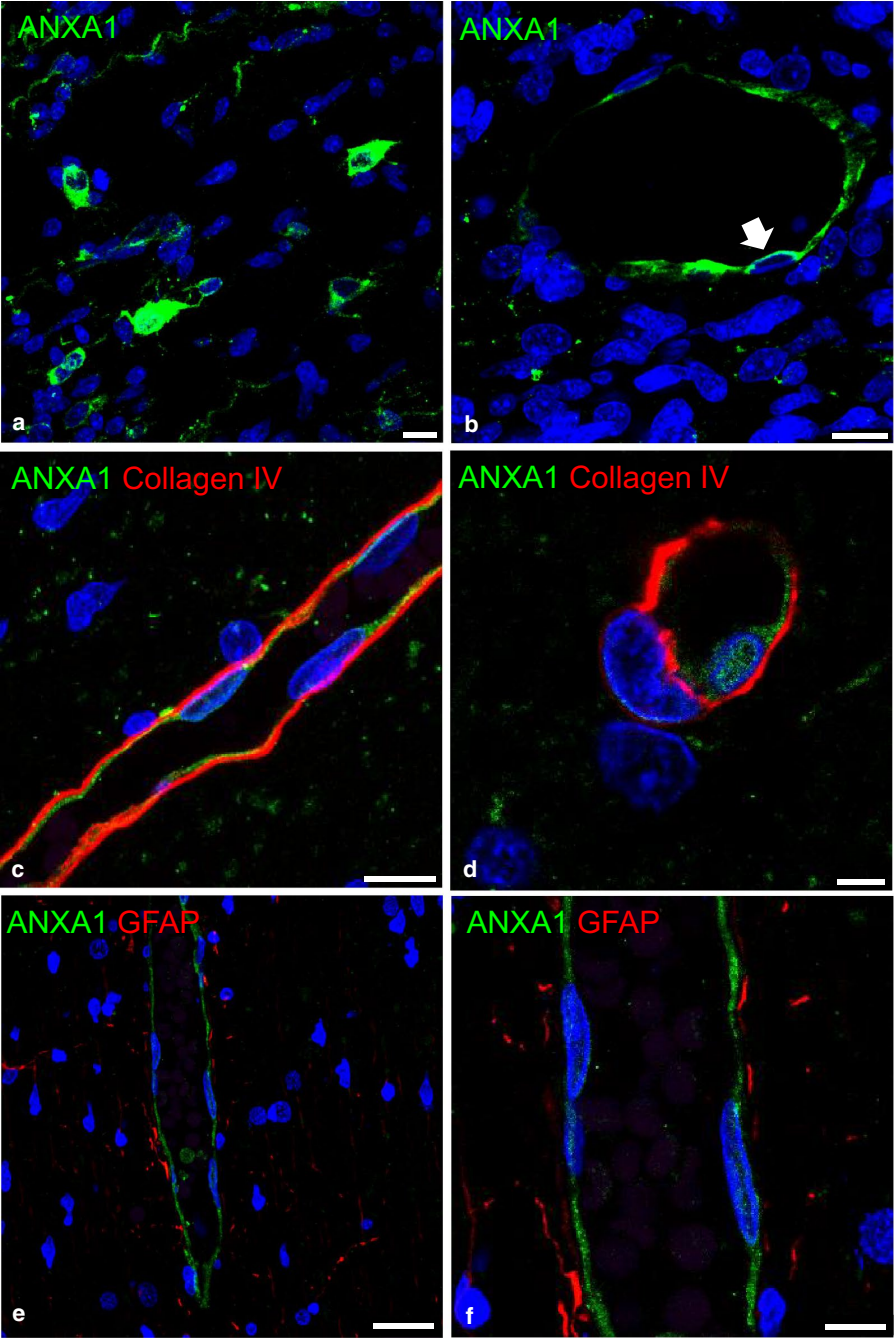
Localisation by immunofluorescence confocal microscopy of ANXA1 in human foetal forebrain at mid-gestation. **a**, **b** Single immunolabelling for ANXA1 (*green*) shows **a** high expression of the protein in microglia-like cells and **b** in venular, endothelial cells. Note in **b** the prevalent expression of ANXA1 on the luminal side of the endothelial plasma membrane (*arrow*). **c**, **d** Double immunolabelling for ANXA1/collagen IV. **c** The vascular basal membrane revealed by collagen IV allow to identify the shows ANXA1 reactivity localised on the endothelial lining; **d** ANXA1-negative pericyte embedded in the basal lamina and the localization of ANXA1 on the endothelial cell nucleus. **e**, **f** Double immunolabelling for ANXA1/GFAP shown on a confocal single optical plane. **e** ANXA1 reactıve endothelial cells in contact with GFAP labelled perivascular astrocyte processes; **f** a detail of ANXA1 localization on the endothelial membranes. Nuclear counterstaining with TO-PRO® 3. *Bars* **a**, **b** 10 μm; **c** 15 μm; **d** 4 μm; **e** 25 µm; **f** 8 µm

The BBB was long considered immature and not fully functional during development, but there is now considerable evidence that this is not completely true, and that many barrier functions are effective from the earliest stages of brain ontogeny [85–87]. While this review does not focus on the development of the BBB, it is interesting to report that we have evidence that ANXA1 is expressed by microglia-like cells and BBB endothelial cells during human foetal development, further supporting a role for the protein in prenatal brain development (Fig. 3). ANXA1 mainly localizes to endothelial cell cytoplasm and plasma membranes (Fig. 3b) and to a lesser extent to the endothelial nucleus (Fig. 3d). It has been suggested that in vitro nuclear translocation of ANXA1 could be induced by mitogenic signals [88] and that it could be a negative prognostic factor in cancer [66, 67, 89]. However, the expression of ANXA1 during normal brain vascularization and BBB differentiation has not been described before and needs to be further investigated.

In contrast, a major feature of aging is a decline in BBB integrity [90], with increased paracellular permeability and decreased TJ protein expression reported in murine models [91]. Intriguingly, human dermal fibroblasts, which share a mesenchymal developmental origin with endothelial cells, exhibit a profound decrease in ANXA1 expression with aging [92], leading us to speculate that endothelial ANXA1 downregulation may be partially responsible for the aged BBB phenotype. This idea is supported by the discovery that ANXA1 null mice have higher, albeit non-pathological, BBB leakage than age-matched wild-type controls [64]. Interestingly a protective role for ANXA1 has been reported in wound healing in the gut [93] and bladder epithelia [94], both of which are characterised by the presence of intercellular tight junctions; structures known to be regulated by ANXA1 in the BBB [80].

## BBB and ANXA1 in human disease

Several studies have indicated a role for ANXA1 in neurological diseases, including Alzheimer’s disease (AD), and stroke; although the most compelling evidence is for an involvement in the pathology of MS.

### Alzheimer’s disease

Alzheimer’s disease is an age-related neurodegenerative disease and the most common form of dementia. Pathologically, it is associated with neuronal loss, and consequent loss of brain volume that is most pronounced around the medial temporal lobe areas, particularly in the hippocampus. Histologically, *post mortem* brains of patients present widespread plaques and neurofibrillary tangles; important factors in AD pathogenesis. The accumulation of amyloid β (Aβ) peptides is believed to be a detrimental factor in Alzheimer’s disease progression, contributing to exacerbated inflammation, microglial activation and neuronal loss. Increased Aβ levels in patients’ brains may result from either Aβ overproduction or inadequate Aβ clearance. Aβ can be cleared via the cerebrospinal fluid (CSF) and interstitial fluid (ISF) and by enzymatic degradation, but an important route for Aβ removal is through efflux transporters present at the brain barriers. [95]. Morphological changes at the brain barriers that occur in healthy aging are accelerated and aggravated in AD. Microvascular reduction and neurovascular dysfunction have been reported in AD [96]. Degeneration of pericytes occurs [96, 97] and thickening of the basal lamina is more pronounced [98].

Human *post mortem* studies of AD have reported upregulated expression of ANXA1 in lesion-associated glia [99, 100] which, given the potent pro-resolution/anti-inflammatory actions of ANXA1 in the periphery [101] may reflect an endogenous attempt to limit cell death. This idea is supported by the actions of ANXA1 in promoting non-phlogistic (non-inflammatory) microglial phagocytosis, even in the face of inflammatory challenge with Aβ [83], and is further supported by the recent identification of a single nucleotide polymorphism in the regulatory region of ANXA1 that associates with susceptibility to AD [102].

Increasing evidence indicates that disruption to the integrity of the blood–brain barrier is a feature of AD [90, 103]. Leakage of circulating plasma components into the brain parenchyma correlates well with both *post mortem* brain staining of AD [104] and with the rate of cognitive decline seen in living AD patients [57, 105]. The impairment of BBB function seen in AD is not restricted to plasma leakage, however, AD-related defects have been reported in cerebral endothelial efflux transporter activity [106, 107] and in abnormal emigration of leukocytes into the neuronal tissue [108, 109]. These challenges to BBB integrity may have significant consequences for AD progression, as there is now strong evidence linking enhanced BBB permeability with impaired cognitive function, including aspects of memory [51], language [52], performance on the mini mental state exam [57] and Oxford handicap scale [54]. Given the role of ANXA1 in controlling BBB TJ formation and particularly in limiting leukocyte extravasation, it is intriguing to speculate on whether the protein plays a role in the microvascular endothelial phenotype of AD and whether application of exogenous protein may be able to limit disease-associated BBB changes. Although this has not been studied directly, raised inflammation and leukocytes trafficking in the peripherally has also reported during disease progression [95].

### Multiple sclerosis

Multiple sclerosis (MS) is an autoimmune inflammatory disease of the central nervous system associated with demyelination and axonal loss, eventually leading to neurodegeneration. In general, it affects people under 50 years old with symptoms usually starting between the ages of 20 and 40. It is a disease with a vast clinical and pathological heterogeneity, but manifests in three principal forms: relapsing remitting (RRMS; the most common form), primary progressive (PPMS) and secondary progressive (SPMS) which tends to be the final stage of RRMS. An important factor in the pathogenesis of MS is the BBB which is compromised during the course of disease [110, 111].

A direct link between BBB impairment, ANXA1 and disease has been indicated in MS, where a clear loss of ANXA1 expression has been identified in the brain parenchymal capillaries of MS patients, distant from lesion sites [80]: a feature which may contribute to the loss of BBB integrity seen in this condition [112]. Importantly, as ANXA1 is also expressed in leukocytes, including both lesional and perivascular macrophages and lymphocytes [113], its role in MS may extend beyond the regulation of inter-endothelial TJs, to directly modulating the autoimmune side of the disease. ANXA1 has long been known to inhibit leukocyte migration [101] through its interaction with the integrin VLA4 [74], closely resembling the main mechanism of action of natalizumab, and highlighting the potential of ANXA1 for therapeutic use. ANXA1 may serve as a checkpoint between leukocytes and the BBB, on the one hand protecting and correcting BBB leakage, and on the other directly controlling leukocyte entry into the brain parenchyma.

### Neurovascular disease and stroke

There is further pre-clinical evidence for a protective/therapeutic role of ANXA1 in the cerebral vasculature in stroke and other neurovascular diseases. Although much work has focussed on the role of ANXA1 as a modulator of inflammatory microglial activity [81], there is evidence for a role in the cerebral vasculature itself. Most notably, administration of human recombinant ANXA1 has been shown to markedly reduce lesion size, clinical score and markers of leukocyte infiltration in murine mid-cerebral artery occlusion models of stroke [114]. Animals lacking the ANXA1 receptor Fpr2/3 showed markedly greater BBB leakage post-ischaemia than their wild-type counterparts [115]. Intriguingly, studies of ischaemic preconditioning regimens, including both chloral hydrate anaesthesia [116] and hypothermia [117] indicate that these protective treatments act at least in part through upregulation of ANXA1.

Given these various clinical and pre-clinical data, we would argue that clinical studies of the pharmacokinetics and pharmacodynamics of ANXA1 in healthy subjects are warranted, as a further step towards evaluating its potential use in patients with MS and other diseases characterised by BBB impairment.

### Estrogen, ANXA1 and the BBB in neurovascular disease

An intriguing aspect of the BBB in neurovascular diseases such as stroke lies in the interactions of estrogen and ANXA1, and the possible role this protein plays in the vasculo-protective action of the hormone. It has long been identified that stroke and other neurovascular diseases show marked sex differences in their incidence, with males being significantly more commonly affected than females [118]. Whilst numerous factors contribute to this difference, including rates of metabolic diseases and environmental influences, the sex steroid hormone estrogen has been shown to be a major discriminating factor [119]. The neuroprotective functions of this hormone have been extensively studied [118], and it is known that it can exert regulatory actions upon immune system function [120], but more recent work suggests that estrogen can directly target the BBB itself. In particular, estrogen has been shown to both protect endothelial cells from cytotoxic stimulation and to directly regulate components of the BBB, including ANXA1, exerting a positive influence on barrier integrity, actions that together help preserve BBB function in the face of inflammatory challenge.

Estrogen is directly protective of cerebral endothelial cells following ischaemic damage in vitro, most notably modelled by deprivation and restitution of oxygen and glucose (OGD/R). Here, the major circulating estrogen, 17β-estradiol, was shown to have a potent cytoprotective action, limiting overt cell death [121], but also acting more directly to prevent hypoxia-inducible factor 1 α (HIF-1α)-mediated down regulation of the TJ molecules occludin and claudin-5 [122, 123]. These protective effects of estradiol on cell viability could be replicated by the estrogen receptor α (ERα) agonist propylpyrazoletriol (PPT) [121], whilst the actions on TJs were mimicked by the ERβ agonist diarylpropionitrile (DPN) [122]. These findings highlight an important aspect of the actions of estrogen upon the BBB, namely the complexity of receptor-mediated signalling, as brain endothelial cells have been shown to express all three major estrogen receptors, ERα, ERβ and the G-protein coupled estrogen receptor 1 (GPER) [124–126]. A major downstream outcome of OGD/R damage is the induction of reactive oxygen species and, as has been repeatedly shown in studies of the neuroprotective potential of estrogen, the hormone is able to exert a powerful antioxidant, vasculoprotective effect [127]. Estrogen has been shown to protect cerebral endothelial cells from both OGD/R-induced [121] and iron-mediated oxidative stress [128], although studies differ on the relative importance of ERα and ERβ.

Protection against oxidative stress appears to be a relatively general effect of estrogen [129], but there is evidence that it can additionally exert BBB-specific protective actions, most notably through modulation of inter-endothelial tight and adherens junction protein expression. We, and others have shown estradiol to upregulate expression of the key TJ molecules claudin-5 [130], occludin and zona occludens-1 [64, 131], and importantly to induce the intracellular relocation of these molecules to the cytoplasmic membrane. Whilst estrogen appears to directly regulate claudin-5 at a transcriptional level [125], the actions of the hormone upon occludin and ZO-1 are mediated through ANXA1 [64] following activation of ERβ. Fewer studies have been conducted upon the effects of estrogen on the BBB in vivo, but it is known that male mice show significantly enhanced BBB permeability following inflammatory challenge than age-matched females, a protection lost in ovariectomised animals and restored by treatment with estradiol [64].

In addition to preventing small molecule entry into the brain parenchyma, the BBB is an important check on the ability of leukocytes to enter the central nervous system [46]. Inflammatory stimulation can activate the cerebral endothelium and permit entry of leukocytes into, at the least, the perivascular space, primarily through upregulation of cell adhesion molecules such as intercellular adhesion molecule 1 (ICAM-1) and vascular cell adhesion molecule 1 (VCAM-1) [132]. Estrogen, again acting through ANXA1, can counteract these changes, reducing adhesion molecule expression on the luminal surface of the endothelium [64, 133], and ultimately preventing leukocyte adhesion and transmigration in the face of inflammatory challenge [64]. In this case however, the actions of estrogen appear to be mediated by GPER, acting to phosphorylate ANXA1 on its N-terminal serine residues [64], thereby promoting its secretion and autocrine/paracrine feedback [79], ultimately resulting in a down-regulation of ICAM-1 expression.

Although not all effects of estrogen upon the BBB could be considered protective, e.g. estrogen down-regulates expression of the major efflux transporter BCRP via ERβ [134–136], in general the hormone appears to act to counter the damaging impact of peripheral inflammation, protecting the brain from homeostatic challenge. Intriguingly, two of the most important of these actions, namely the preservation of endothelial TJ integrity and the downregulation of inflammatory adhesion molecule expression, appear to be mediated through ANXA1, reinforcing the role of this protein as a central mediator of BBB function.

### Metabolic disease and the BBB

Metabolic diseases, such as obesity and diabetes mellitus, are major and increasing sources of ill health in the human population. These conditions have deleterious effects upon virtually every physiological system, and it is increasingly apparent that the CNS is not spared in this regard. In particular, there is now accumulating evidence for a direct impact of metabolic dysregulation upon the BBB, representing an important pathway through which disorders of metabolism can affect behaviour and cognition [60, 137].

Several studies have showed obesity to be associated with structural brain deficits, including atrophy in the frontal lobes, hippocampus, thalamus [138], white [139] and grey matter [140], increased BBB permeability [141–143] and the remodelling of brain microvessels [144], suggesting a direct link between dietary habits and BBB function. Moreover, obesity has been reported to alter neurovascular unit organization, leading to increased numbers of perivascular microglia [145] and activation of both microglia and astrocytes [145–147]. Interestingly, it was reported that even the offspring of obese mice presented increased BBB permeability at birth, suggesting that maternal gestational obesity may be able to compromise BBB formation during development [146].

Furthermore, obesity-induced BBB disruption has been associated with down-regulation of cytoskeletal component expression in endothelial cells, including vimentin and tubulin [148], and the TJ proteins occludin [142], claudin-5 [142, 149] and ZO-1 [148]. As ANXA1 is an essential regulator of BBB tightness through stabilisation of the cytoskeleton [75], we would speculate that its expression or post-translational modification might also be affected in obesity.

Similarly, experimental studies have also shown that obesity is associated with increased neuronal death, BBB leakage [141], and infarct volume [144, 150, 151] following induction of an ischaemic episode. The deleterious effects of obesity in experimental models of stroke may be mediated, at least partly, through activation of matrix metalloproteinase (MMP)-9, as high fat diet-induced obesity increases MMP-9 expression in ischaemic murine brain [144, 150] and MMP-9 knockdown reversed the damaging effects of obesity following ischaemic challenge [144].

Moreover, there are few if any, direct studies of the mechanisms underlying the deleterious influence of obesity upon the BBB, but inflammatory pathways may well play an important role. It is increasingly evident that white adipose tissue secretes a wide variety of biologically active adipokines [152], including both pro-inflammatory (leptin, tumor necrosis factor α (TNFα) and interleukin 6 (IL-6)) and anti-inflammatory (adiponectin) factors, which have the potential to regulate endothelial function [153], and intriguingly plasma ANXA1 levels inversely correlate with indicators of abdominal visceral fat [154], suggesting that loss of endothelial ANXA1 may also occur in response to chronic obesity-driven inflammation. Obesity has been associated with activation of pro-inflammatory pathways in the brain, as a high fat diet up-regulated expression of toll like receptor 4 (TLR4), high-mobility group protein B1 (HMGB1), vascular endothelial growth factor (VEGF) and COX-2 [155]. In addition, db/db mice, constitutively showed perivascular macrophage infiltration [142], exacerbated nuclear factor κ B (NFκB) activation [156], and increased IL-1β, IL-6, monocyte chemoattractant protein 1 (MCP-1) and TNFα release [142]. How and if ANXA1 is involved in such pathways remains speculative, but our preliminary data suggests that ANXA1 null mice exhibit greater cerebral perivascular CD45+ cell accumulation in response to diet-induced obesity than their wild-type counterparts, indicating a role for the protein in the regulation of immune cell entry into the brain, and further supporting our hypothesis that ANXA1 acts to protect BBB integrity. Furthermore, the finding that low levels of oxygen inhibit the expression of ANXA1 in the pre-adipocytes suggest that ANXA1 may have a role in the regulation of inflammatory and pro-resolvin pathways necessary to restore homeostasis in the inflamed adipose tissue [154].

### Diabetes mellitus

A major long-term complication of obesity is diabetes mellitus (DM). A growing body of clinical evidence suggests that DM is associated with neuronal dysfunction; *post mortem* human studies indicate that patients with DM exhibit reduced brain volume in both grey and white matter [157–159] most notably in the hippocampus [157, 160]. The importance of these findings is emphasised in parallel studies indicating DM as a major risk factor for dementias including AD [161, 162] and mild cognitive impairment [159, 161], and for stroke [163, 164]. A variety of potential mechanisms have been identified for these connections, but the role of the BBB has been somewhat under-investigated despite accumulating evidence for its involvement in the neuronal component of DM. Circulating level of ANXA1 have been reported to be downregulated in DM [165], data we have confirmed in our high fat diet animal model (ES, unpublished data).

DM-induced BBB disruption is associated with alterations in neurovascular unit organization, with experimental diabetic models exhibiting marked reduction in numbers of pericytes [166], but with microglial [167, 168] and astrocytic activation [168, 169], indicative of a local inflammatory response. Additionally, exposure of endothelial cells to hyperglycaemia induces a down-regulation in expression of tight TJ occludin [170–172], claudin 5 [149, 170, 172] and ZO-1 [171]. Diabetes mellitus (type 1 diabetes-T1DM) has been associated with changes in the endothelial basal lamina, further contributing to disrupted BBB permeability [169].

The alterations in BBB permeability induced by disruptions to glucose homeostasis may be driven, at least in part, by increased activity of matrix metalloproteinases (MMPs). Studies have shown exacerbation of MMP-2 activity in serum from patients with T1DM [173], from STZ-induced diabetic animals [167, 171] and from rat models of diabetes mellitus type 2 (T2DM) [174]. Moreover, in vitro studies show that hyperglycaemia can increase MMP2 activation [175], whilst BBB disruption induced by advanced glycation end-products (AGEs) produced under hyperglycaemic conditions can be reversed by inhibiting MMP-2 activity [176]. Streptozotocin (STZ)-induced diabetic mice present BBB disruption associated with exacerbated MMP-9 activity, while treatment with S-nitrosoglutathione, a nitric oxide modulator which is protective against oxidative/nitrosative stress, reduces MMP-9 activity and restores normal BBB permeability [177]. Intriguingly, ANXA1 has been both positively and negatively associated with MMPs expression in cancer [178–180], and has been shown to be the target of AGE-dependent non-enzymatic glycosylation in pulmonary endothelial cells in STZ-induced T1DM [181], suggestive of a link between AGE and BBB breakdown in DM.

**Fig. 4:**
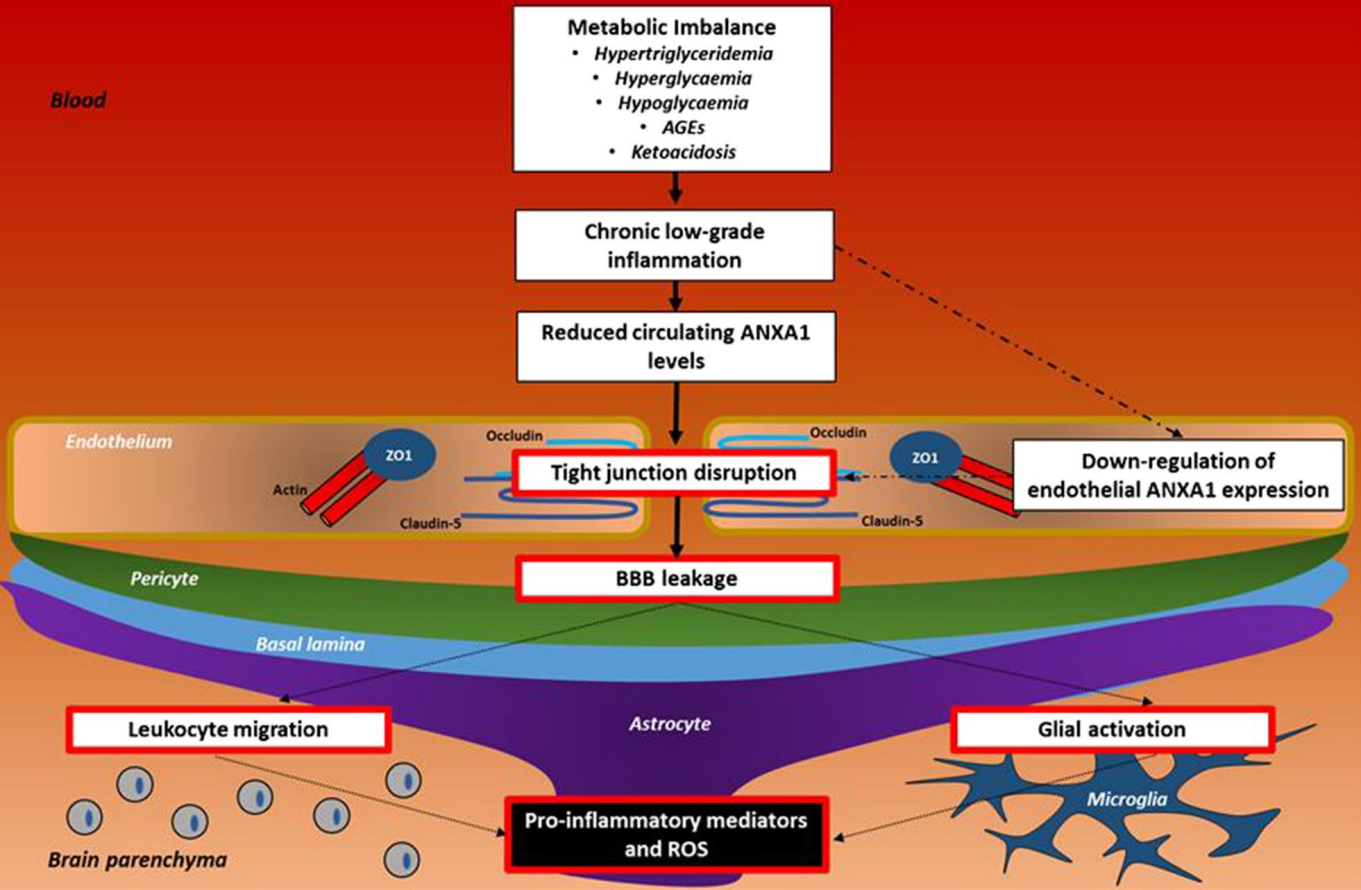
Schematic representation of possible mechanisms linking metabolic disorders, BBB dysfunction and neurodegeneration. Metabolic imbalance associated with obesity and DM leads to chronic systemic, low-grade inflammation and the down-regulation of circulating ANXA1. Interaction of circulating soluble factors with brain endothelial cells induces tight junction disruption and BBB leakage, permitting leukocyte migration and glial activation, which in turn can impair neuronal function through production of pro-inflammatory mediators and reactive oxygen species (*AGEs* advanced glycation end-products; *ROS* reactive oxygen species)

Beyond the DM-induced alterations of BBB function described above, DM has also been associated with CNS infiltration of bone marrow-derived macrophages and raised levels of pro-inflammatory cytokines in the brain parenchyma [167, 169], indicating that the immunological barrier functions of the BBB are similarly disrupted by DM. Furthermore white matter lesions, lacunar infarcts, small strokes, and reductions in cerebral blood flow are also reported being induced by DM (type 1 and 2) [182, 183].Together, these results suggest that DM-induced effects on BBB function depends on the interaction between several conditions (hyperglycaemia, hypoglycaemia, AGEs) inherent to the pathology (Fig. 4). The possible involvement of ANXA1 in mediating/protecting against these changes has not been investigated to date, but it is known that human patients with T2DM exhibit reduced serum levels of ANXA1 compared with healthy control individuals [165]. If this decline is reflected in ANXA1 expression in the BBB itself, we can speculate that ANXA1 loss would play a major role in transmission of inflammatory stimuli into the brain parenchyma, and the associated cognitive disturbances seen in DM [60] (Fig. 4).

## Conclusion

The BBB as a critical communication interface between the brain and the rest of the body has long been known to play a role in neurological disease, but it is increasingly realised that dysfunction is a feature of other homeostatic disorders, most notably metabolic diseases such as obesity and diabetes mellitus. A loss of BBB integrity is thought to directly contribute to the cognitive, behavioural and neurological symptoms of these conditions, which to date have received scant attention in either research or medical practice. Strategies to reverse BBB damage may therefore be of great utility in addressing the neurological symptoms of many clinical conditions. In this review, we propose that therapeutic use of a major regulator of BBB integrity and function, ANXA1, may serve as such a strategy. Several alternative approaches to the therapeutic use of ANXA1 are currently under development. One aims to avoid the inherent problems of using a full-length protein in the clinic; namely the administration of N-terminal ANXA1 peptides [115, 184]. Unfortunately these efforts are somewhat limited by the propensity of these agents to signal through both FPR2 and the pro-inflammatory receptor FPR1 [185]. More promising might be through the delivery of microvesicle-encapsulated proteins. This latter approach has been shown to be potently anti-inflammatory in animal models of rheumatoid arthritis [186], colitis [93] and atherosclerosis [187]. Hence it could prove to be valuable in the treatment of conditions characterised by damaged BBB integrity, including such major disorders as Alzheimer’s disease, multiple sclerosis and diabetes mellitus. Delivery of ANXA1 thus holds great potential as a way to reverse BBB damage induced by inflammation or metabolic challenge, and in doing so restore normal BBB function.

## Abbreviations

ABC: ATP-binding cassette transporters
Aβ: amyloid beta
AD: Alzheimer’s disease
AGE: advanced glycation end products
ANXA1: annexin A1
BBB: blood–brain barrier
BCRP: breast cancer resistance protein
CNS: central nervous system
COX2: cyclooxygenase 2
CSF: cerebrospinal fluid
DM: diabetes mellitus
DPN: diarylpropionitrile
ER: estrogen receptor
ERK: extracellular signal-regulated protein kinase
FPR2: human formyl peptide receptor 2
Fpr2/3: murine FPR2 equivalent
GFAP: glial fibrillary acidic protein
GPER: G-protein coupled estrogen receptor 1
GTPase: guanosine triphosphate hydrolase
HIF-1α: hypoxia-inducible factor 1 α
HMGB1: high-mobility group protein B1
ICAM-1: intercellular adhesion molecule 1
IL-6: interleukin-six
ISF: interstitial fluid
LPS: lipopolysaccharides
MAP kinase: mitogen-activated protein kinase
MCP-1: monocyte chemoattractant protein 1
miR-155: micro RNA 155
MMP: matrix metalloproteinase
MRP: multidrug resistance-associated protein
MS: multiple sclerosis
NFκB: nuclear factor κ B
NVU: neurovascular unit
PPMS: primary progressive multiple sclerosis
PPT: propylpyrazoletriol
OGD/R: oxygen and glucose deprivation and restitution
RRMS: relapsing remitting multiple sclerosis
SMPS: secondary progressive multiple sclerosis
STZ: streptozotocin
T1DM: type 1 diabetes mellitus
T2DM: type 2 diabetes mellitus
TLR4: toll like receptor 4
TNFα: tumor necrosis factor α
TJ: tight junction
TO-PRO® 3: red fluorescent nuclei stain
VCAM-1: vascular cell adhesion molecule 1
VEGF: vascular endothelial growth factor
ZO: zona occludens
